# Supplementary material for: Influence of Lipoxygenase Inhibition on Glioblastoma Cell Biology
Source: Int J Mol Sci. 2020 Nov 9;21(21):8395. doi: 10.3390/ijms21218395 (PMC7664864; doi:10.3390/ijms21218395)
Supplement: Supplementary file 1 [file ijms-21-08395-s001.zip › Full Gel.Membrane Images.pdf]

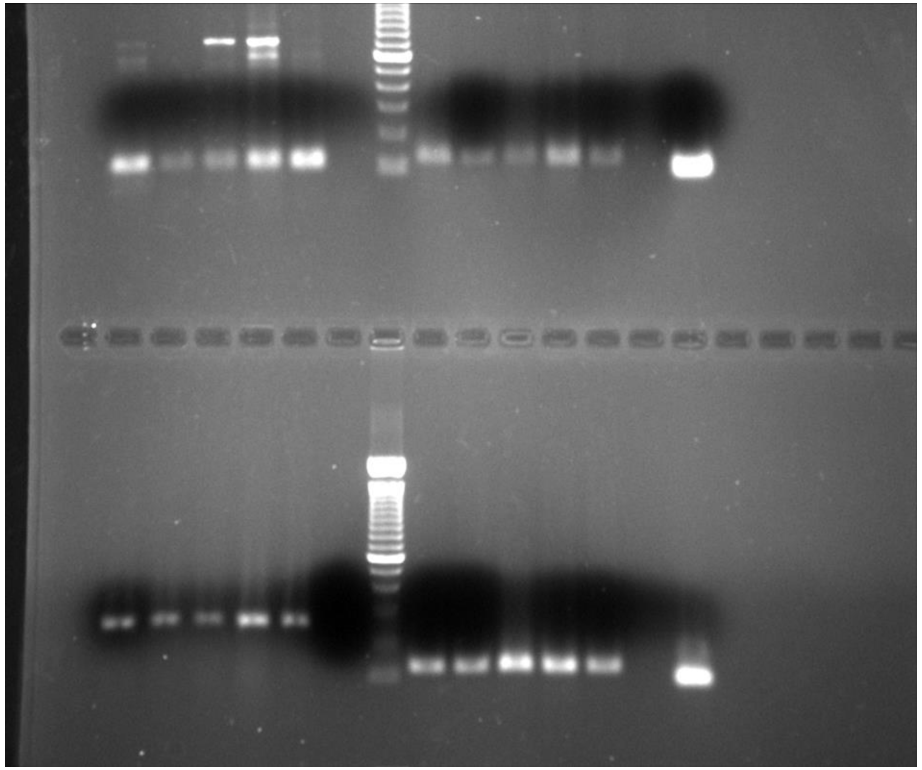

Figure 2B- Top left 15LOX1; Top right 15LOX2; Bottom left GRP132; *Bottom right another gene and is not relevant.*

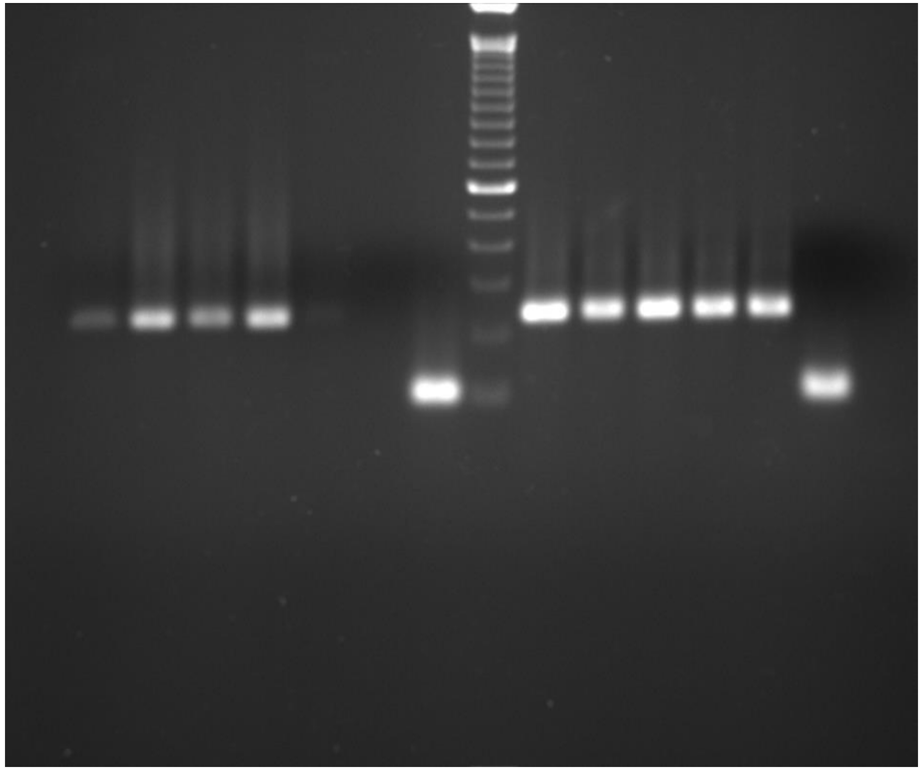

Figure 2B PPAR Gamma and PPAR Beta/Delta

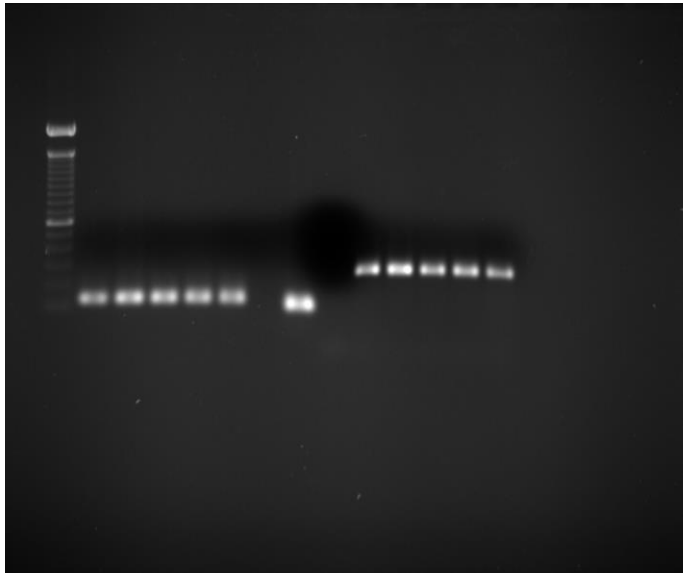

Figure 7A Left/Center

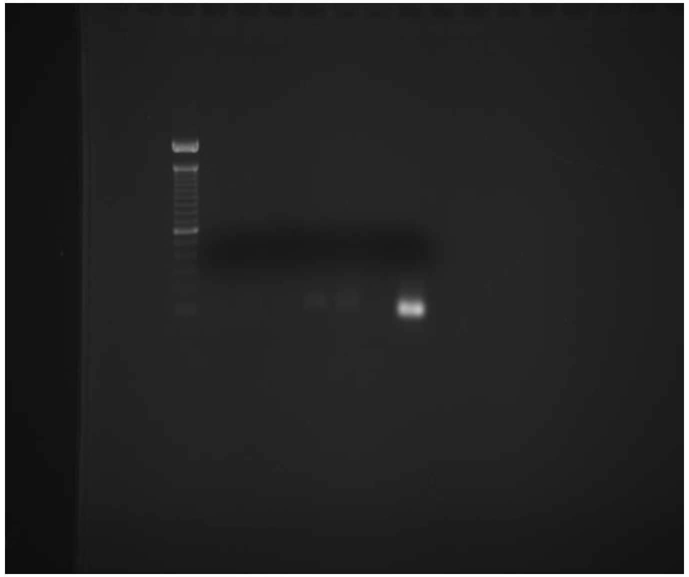

Figure 7A Right

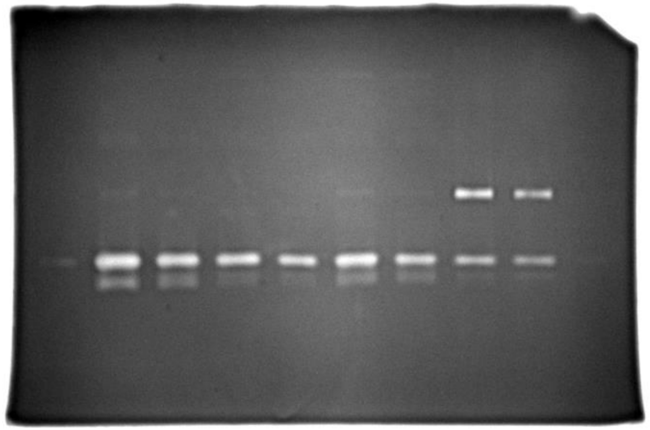

Figure 7E

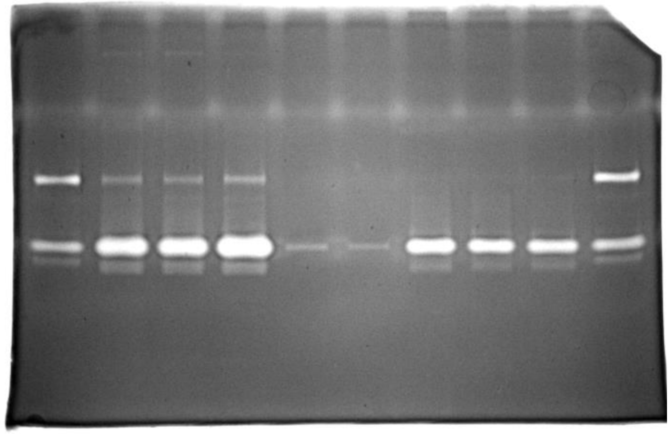

Figure 7F – Right half of the gel. *The left side of gel is another cell line and is not relevant.*

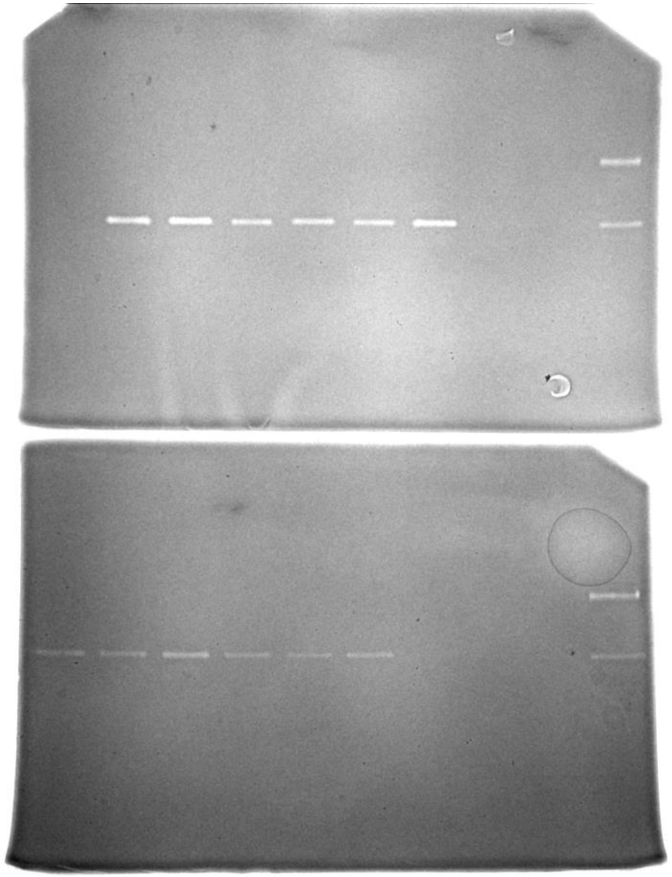

Figure 7H

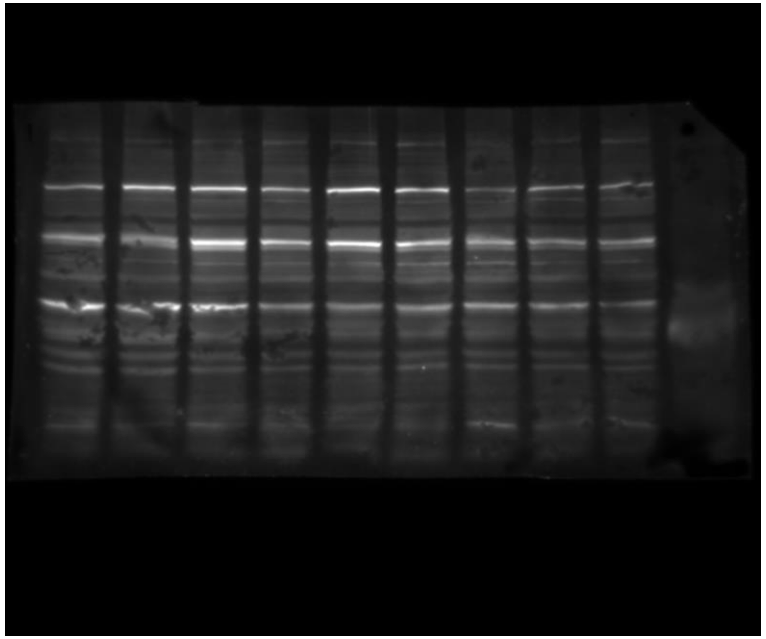

Figure 8A Run1. 15-LOX

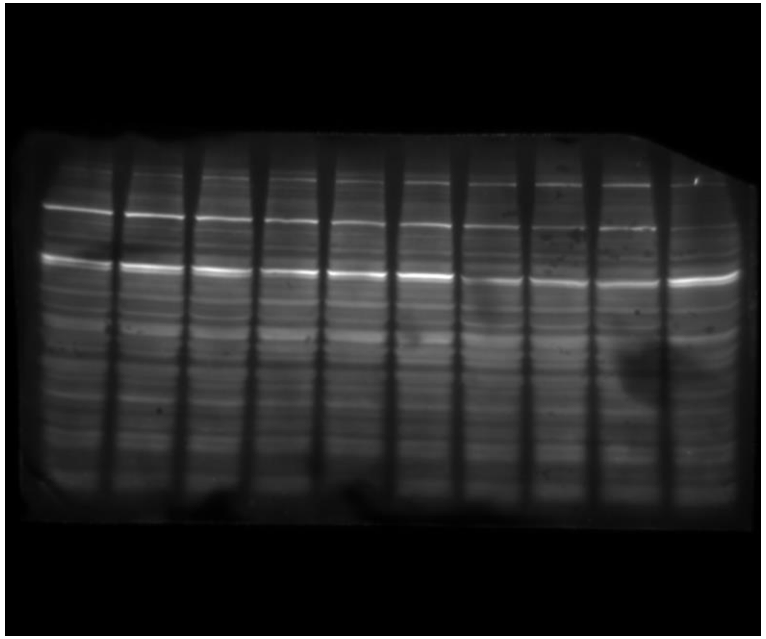

Figure 8B Run1. 15-LOX

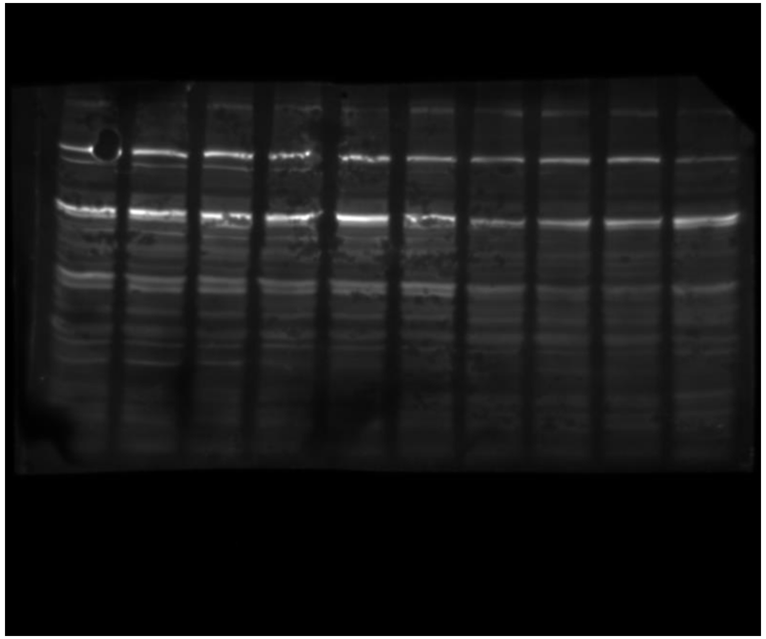

Figure 8B Run2. 15-LOX

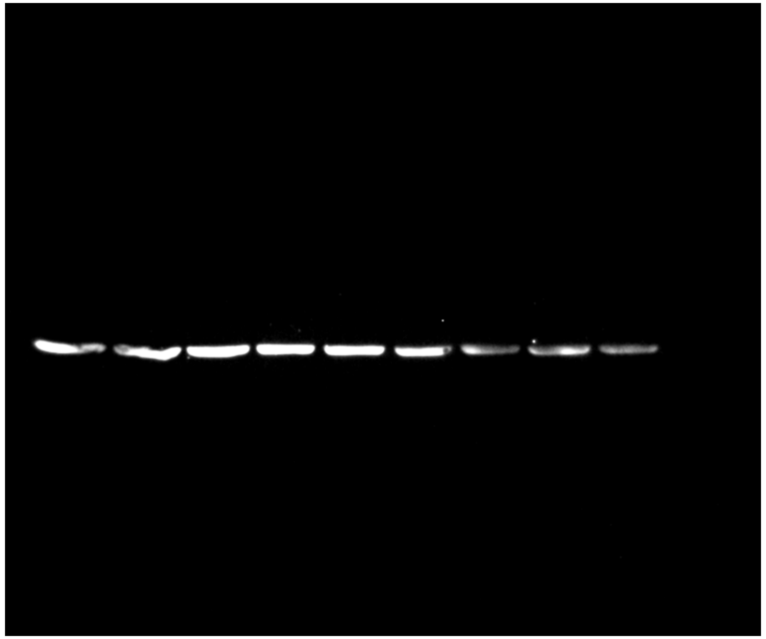

Figure 8A Run1. B-actin

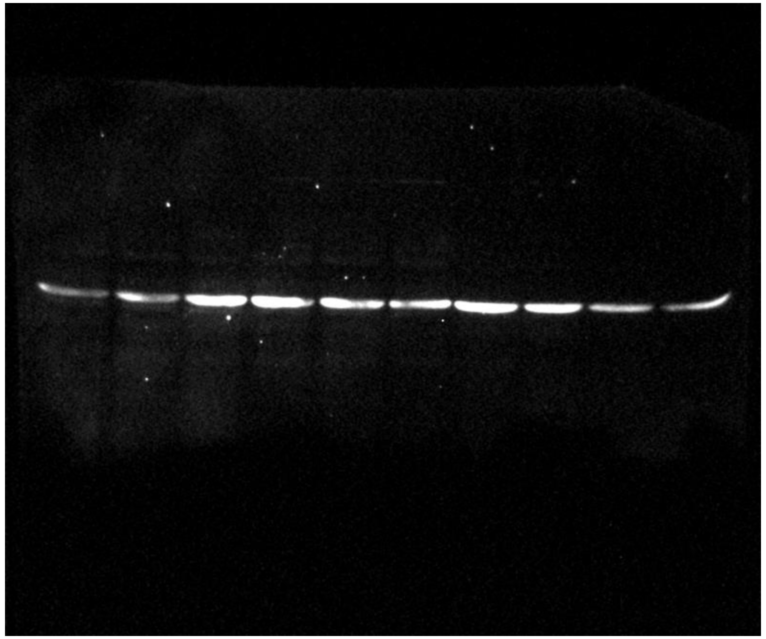

Figure 8B Run1. B-actin

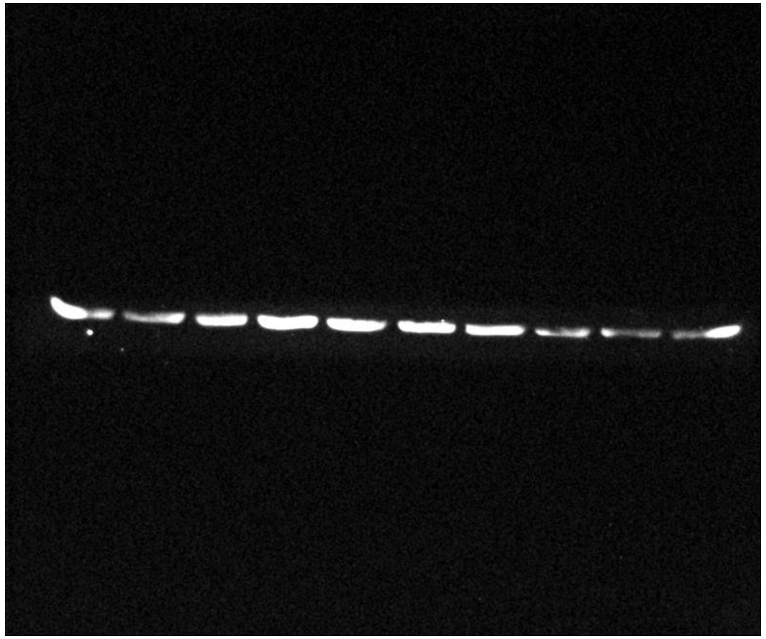

Figure 8B Run2. B-actin
