# Supplementary material for: Influence of Lipoxygenase Inhibition on Glioblastoma Cell Biology
Source: Int J Mol Sci. 2020 Nov 9;21(21):8395. doi: 10.3390/ijms21218395 (PMC7664864; doi:10.3390/ijms21218395)
Supplement: Supplementary file 1 [file ijms-21-08395-s001.zip › Supplementary Figures - Influence of lipoxygenase inhibition on glioblastoma cell biology - 2020.pdf]

Supplementary Material

LIPOXYGENASE PROFILE, ACTIVITY AND MODULATION IN GLIOBLASTOMA CELL LINES

Felipe da Costa Souza¶*, Matthew Thomas Ferreira¶, Alison Colquhoun

Department of Cell and Developmental Biology, Institute of Biomedical Sciences, University of São Paulo, São Paulo, Brazil.

¶ These authors contributed equally to this work and share co-first authorship.

*Corresponding author: f.dcsouza@gmail.com

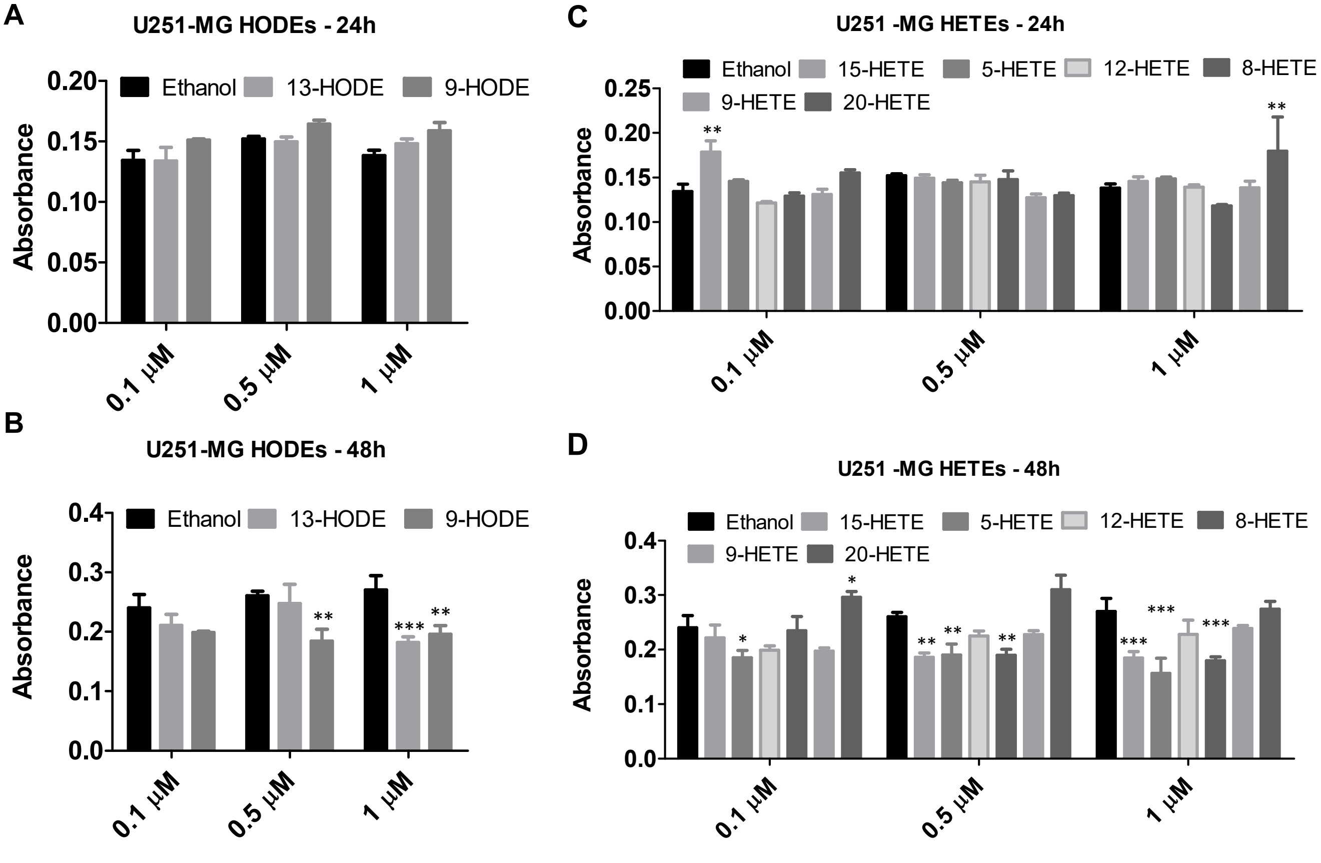

Supplementary 1. Cell viability of U251-MG treated with exogenous lipids by MTT (A, B, C, D). Histograms show MTT absorbance after 24 and 48 hours of treatment with a HODE (A and B) and HETE (C and D) panel. Significance was found only for some of treatments, mostly at 48h. $p<0.05$ (*), $p<0.01$ (**), $p<0.001$ (***). N = 3, in duplicate.

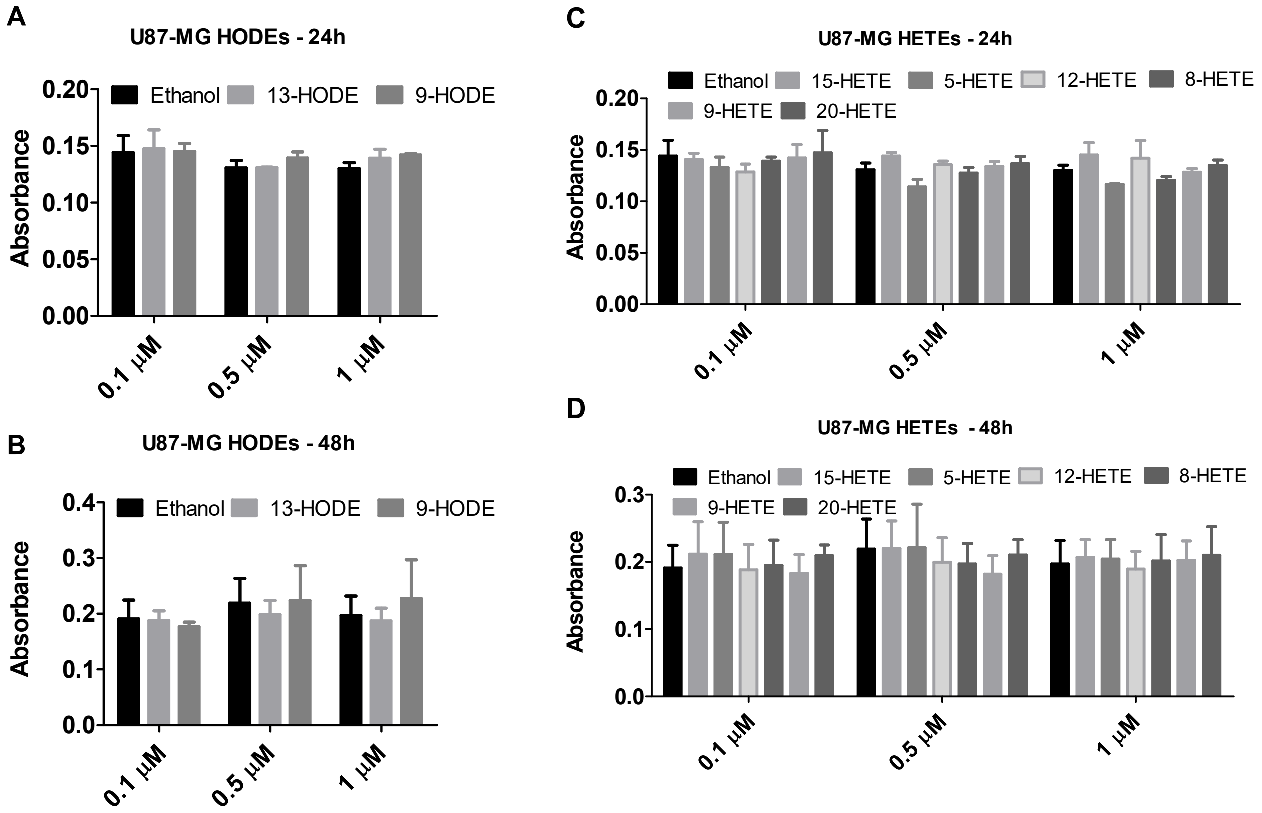

Supplementary 2. Cell viability of U87-MG treated with exogenous lipids by MTT (A, B, C, D). Histograms show MTT absorbance after 24 and 48 hours of treatment with a HODE (A and B) and HETE (C and D) panel. No significance was found with any of the treatments. N = 3, in duplicate.

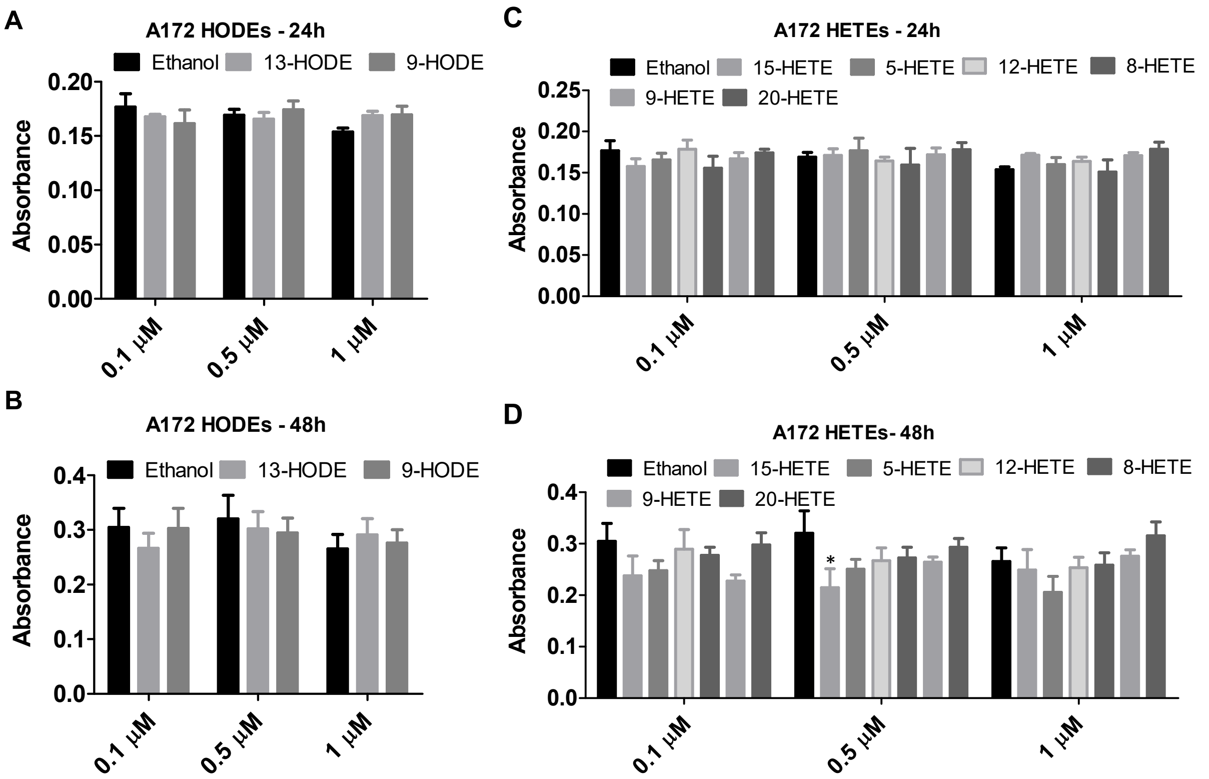

Supplementary 3. Cell viability of A172 treated with exogenous lipids by MTT (A, B, C, D). Histograms show MTT absorbance after 24 and 48 hours of treatment with a HODE (A and B) and HETE (C and D) panel. No significance was found with any of the treatments. $p<0.05$ (*). N = 3, in duplicate.

U251-MG

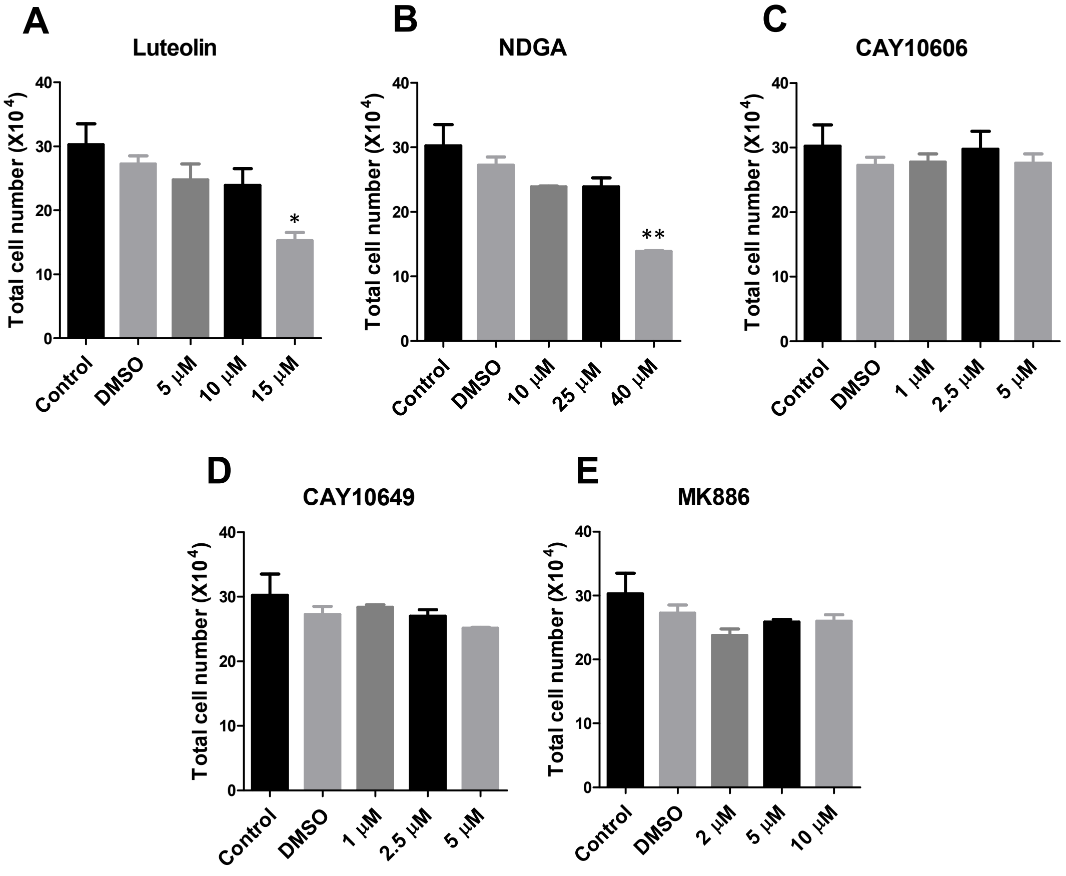

Supplementary 4. Cell counts of U251-MG treated with Luteolin, NDGA, CAY10606, CAY10649, MK886, CAY10678 and CAY10526 at different concentrations (A, B, C, D and E). Graphs show cell number after 72 hours, with the data analysis in relation to the control. No significance was found with any of the 5-LOX inhibitors. Both Luteolin and NDGA reduced cell population at higher concentrations (A and B). $p<0.05$ (*), $p<0.01$ (**), $p<0.001$ (***). N = 3, in duplicate.

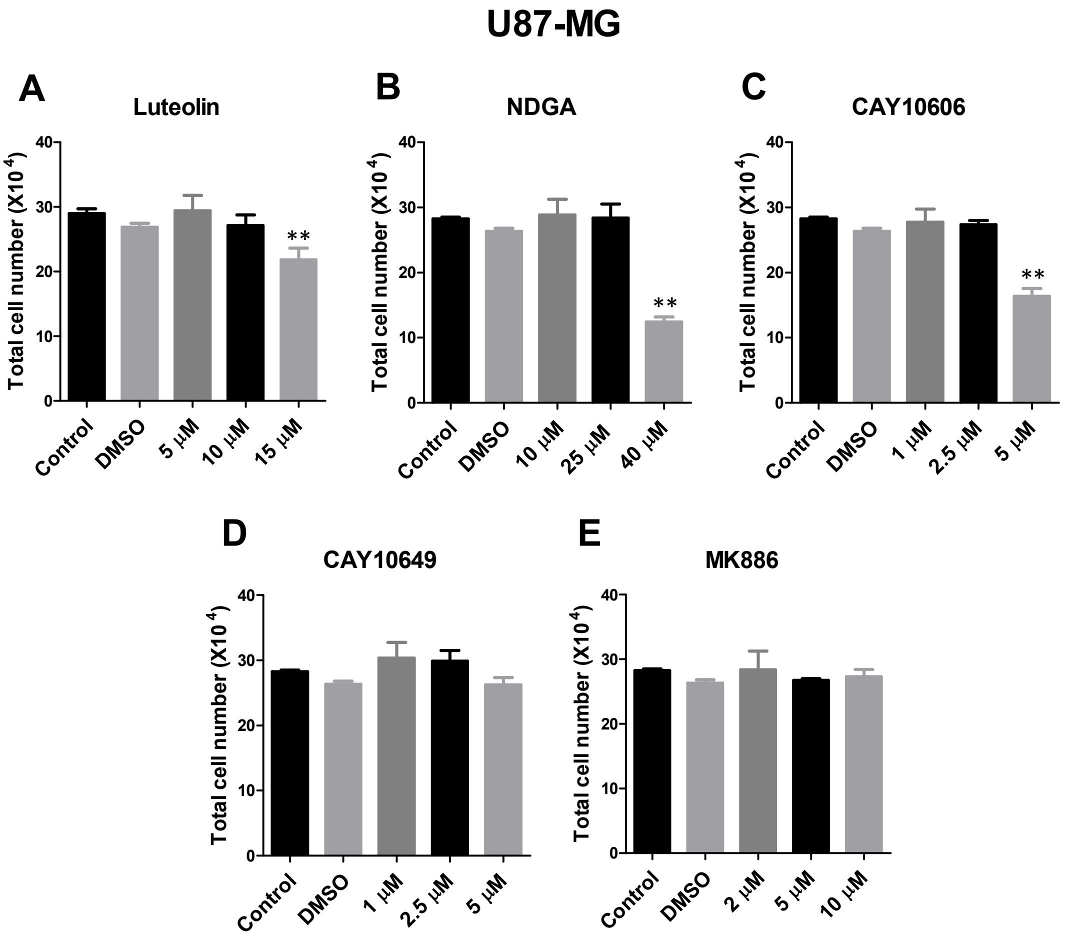

Supplementary 5. Cell counts of U87-MG treated with Luteolin, NDGA, CAY10606, CAY10649, MK886, CAY10678 and CAY10526 at different concentrations (A, B, C, D and E). Histograms show cell number after 72 hours, with the data analysis in relation to the control. No significance was found with any of the 5-LOX inhibitors, except CAY10606 (C). Both Luteolin and NDGA reduced cell population at higher concentrations (A and B). $p<0.05$ (*), $p<0.01$ (**), $p<0.001$ (***). N = 3, in duplicate.

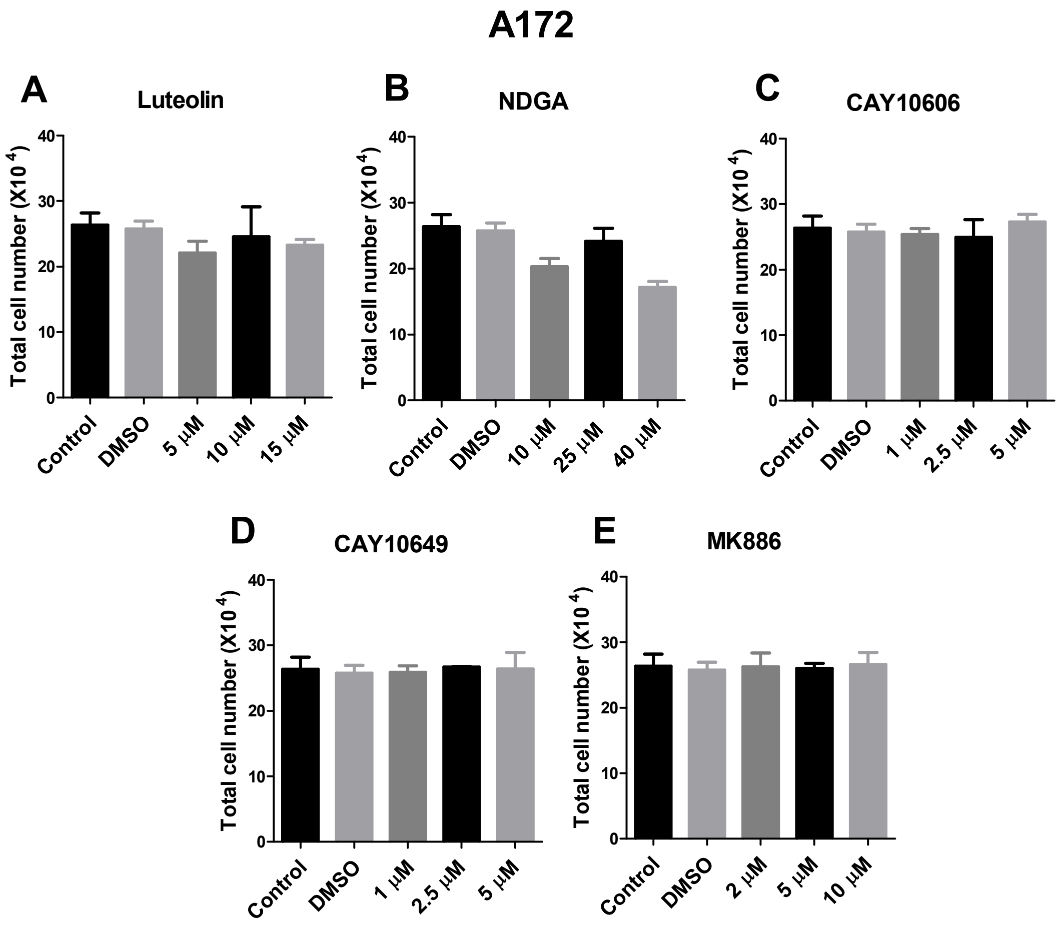

Supplementary 6. Cell counts of A172 treated with Luteolin, NDGA, CAY10606, CAY10649, and MK886 at different concentrations (A, B, C, D and E). Histograms show cell number after 72 hours, with the data analysis in relation to the control. No significance was found with any of the inhibitors. N = 3, in duplicate.

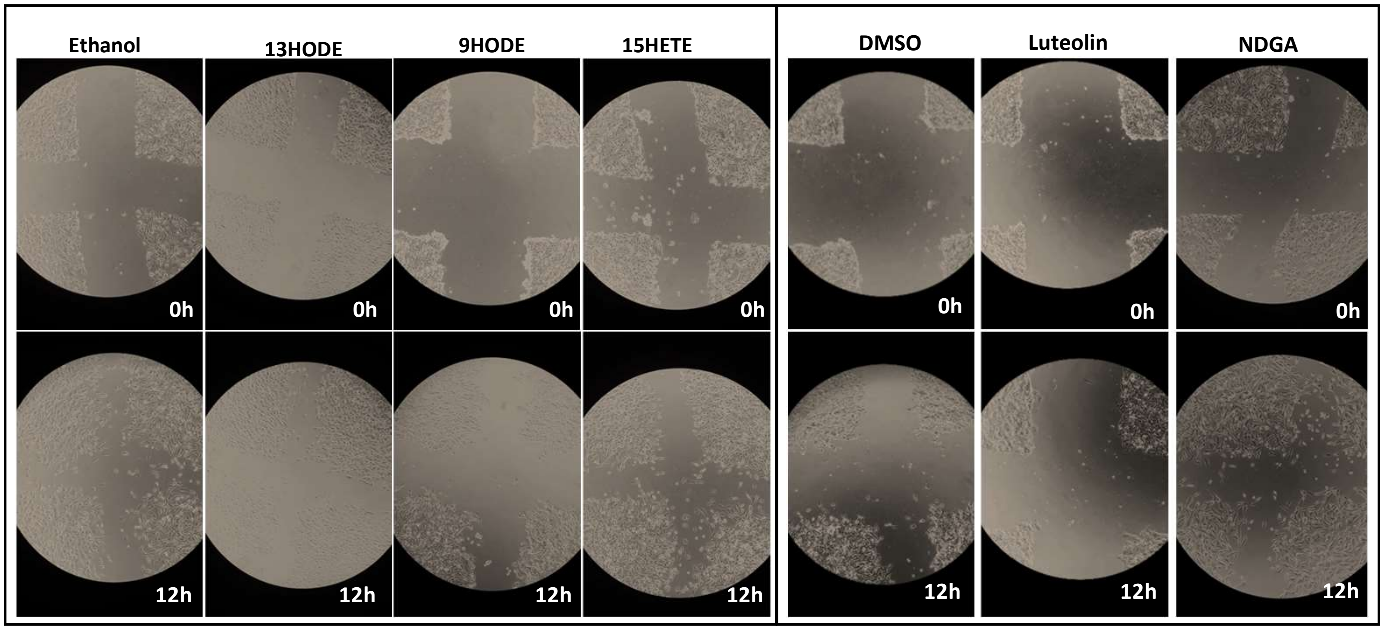

Supplementary Figure 7. Representative images of wound healing assay of cells treated with 13-/9-HODE/15-HETE and their respective controls at 0 h and 12 h, and representative photos of Luteolin/NDGA and their controls at 0 h and 12 h. N=3.
